# Comparison of Anatomical and Non‐Anatomical Resection in Low Microvascular Invasion Risk Solitary Hepatocellular Carcinoma ≤ 5 cm

**DOI:** 10.1002/ags3.70157

**Published:** 2025-12-26

**Authors:** Norifumi Iseda, Shinji Itoh, Kyohei Yugawa, Shohei Yoshiya, Takashi Motomura, Takeo Toshima, Tomoharu Yoshizumi

**Affiliations:** ^1^ Department of Surgery and Science, Graduate School of Medical Sciences Kyushu University Fukuoka Japan

**Keywords:** anatomical resection, hepatocellular carcinoma, intrahepatic metastasis, microvascular invasion, non‐anatomical resection

## Abstract

**Background:**

The role of anatomical resection (AR) in hepatocellular carcinoma (HCC) remains controversial, particularly for tumors ≤ 5 cm without vascular invasion. We aimed to evaluate the long‐term outcomes of AR versus non‐anatomical resection (NAR) in solitary HCC presumed negative for microvascular invasion (MVI) and intrahepatic metastasis (IM).

**Methods:**

We retrospectively analyzed 303 patients with solitary HCC who underwent hepatectomy between 2002 and 2019. Predictive factors for MVI and IM were identified, and 214 patients with solitary HCC ≤ 5 cm and predicted absence of MVI and IM were further analyzed. We compared the perioperative and oncological outcomes between the AR (*n* = 94) and NAR (*n* = 120) groups. Subsequently, we conducted propensity score matching (*n =* 41 per group) and performed subgroup analyses based on tumor size.

**Results:**

Des‐γ‐carboxy prothrombin > 150 mAU/mL was identified as an independent predictor for MVI and IM. Compared with the NAR group, the AR group had a significantly longer operative time, greater blood loss, and higher rate of complications, but showed no significant differences in recurrence‐free survival and overall survival. Recurrence‐free survival and overall survival remained comparable between the two groups after propensity score matching. Subgroup analyses by tumor size (0–2.0 and 2.1–5.0 cm) showed no prognostic advantage for AR over NAR.

**Conclusions:**

For solitary HCC ≤ 5 cm without predicted MVI and IM, NAR and AR result in comparable long‐term outcomes. The resection strategy should prioritize remnant liver function over anatomical extent.

## Graphical abstract

We evaluated whether anatomical resection (AR) provides an oncological benefit over non‐anatomical resection (NAR) in solitary hepatocellular carcinoma ≤ 5 cm without predicted microvascular invasion or intrahepatic metastasis, identified by preoperative DCP ≤ 150 mAU/mL. In this low‐risk cohort, AR did not improve recurrence‐free or overall survival compared with NAR, suggesting that parenchyma‐preserving NAR is an acceptable surgical option for appropriately selected patients.

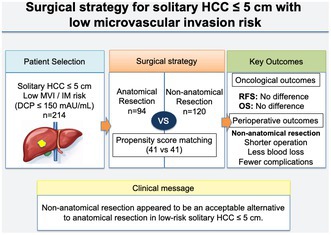

**Abbreviations**
AFP: α‐fetoprotein
AR: anatomical resection
CI: confidence interval
DCP: des‐γ‐carboxy prothrombin
HBsAg: hepatitis B surface antigen
HCC: hepatocellular carcinoma
HCVAb: hepatitis C virus antibody
HR: hazard ratio
IM: intrahepatic metastasis
MVI: microvascular invasion
NAR: non‐anatomical resection
OS: overall survival
RFS: recurrence‐free survival

## 1 Introduction

Hepatocellular carcinoma (HCC) is one of the most common malignancies worldwide [1], and hepatic resection remains a potentially curative option for patients with resectable tumors [2]. Anatomical resection (AR), which involves removal of the entire portal territory containing the tumor, has been widely performed to eliminate possible microvascular invasion (MVI) and intrahepatic metastasis (IM) through the portal venous flow. However, the superiority of AR over non‐anatomical resection (NAR), which preserves more of the liver parenchyma, remains controversial, particularly for solitary HCC without MVI.

As the etiology of liver cancer has shifted in recent decades due to the decline in viral hepatitis and the rise in non‐viral liver diseases, surgical strategies have evolved. Furthermore, advances in laparoscopic liver resection, improved perioperative care, and the emergence of systemic therapies have broadened the treatment landscape [3, 4]. In this context, the oncological necessity of AR for small solitary HCCs with preserved liver function is being reevaluated.

Based on the Japanese Expert Consensus 2023, solitary HCC without MVI is defined as resectable [5]. Accordingly, for the present analysis, we aimed to identify a subgroup of patients who were presumed to meet these criteria, by focusing on patients with solitary tumors and low predicted risk of MVI and IM. Meanwhile, in our previous study, we demonstrated that tumor size > 5 cm was significantly associated with an increased risk of recurrence after hepatic resection [6]. Therefore, we limited the analysis to patients with solitary HCC ≤ 5 cm and low predicted risk of MVI and IM to ensure clinical consistency with both the consensus classification and our prior findings.

Against this background, the present study aimed to compare the perioperative outcomes and long‐term prognosis between AR and NAR in patients with solitary HCC ≤ 5 cm who were presumed to be free of MVI and IM, using both conventional comparative and propensity score matching analyses.

## 2 Methods

### 2.1 Patients

This retrospective study included 303 patients with solitary HCC who underwent hepatic resection at Kyushu University Hospital between January 2002 and December 2019. Patients with extrahepatic disease, tumor thrombus, or insufficient clinical data were excluded. Within the cohort, we identified 176 patients with solitary HCC ≤ 5 cm who were presumed negative for MVI and IM based on the preoperative tumor marker profiles. The surgical procedures for hepatic resection were described previously [2, 7].

### 2.2 Follow‐up Strategy

After hospital discharge following the surgery, all patients underwent monthly screening for recurrence by computed tomography and tumor marker evaluation every 3 months. If recurrence was suspected, magnetic resonance imaging was performed. Overall survival (OS) was defined as the time from hepatic resection to the date of death from any cause. Recurrence‐free survival (RFS) was defined as the time from hepatic resection to the date of first recurrence after resection.

### 2.3 Statistical Analysis

Data were expressed as the median (interquartile range). Statistical analyses were performed using JMP Pro 15 software (SAS Institute, Cary, NC, USA). The Shapiro–Wilk test was used to determine whether continuous variables were normally distributed. Continuous variables were compared using the Mann–Whitney *U*‐test. Categorical variables were presented as the percentage and compared using the chi‐square test or Fisher's exact test. Cumulative RFS and OS rates were calculated using the Kaplan–Meier method, and differences between the survival curves were evaluated using the log‐rank test. Survival data were used to establish a univariate Cox proportional hazards model. Cut‐off values were determined by receiver‐operating characteristic curve analysis. Covariates that were significant at *p* < 0.05 in the univariate model were included in a multivariate Cox proportional hazards model.

## 3 Results

### 3.1 Predictive Factors for MVI and IM

We analyzed 303 patients with solitary HCC who underwent hepatic resection at our institution between January 2002 and December 2019 to identify preoperative factors associated with the presence of MVI and IM (Table S1). Receiver‐operating characteristic curve analyses were performed to determine the optimal cut‐off values for serum α‐fetoprotein (AFP) and des‐γ‐carboxy prothrombin (DCP), which were found to be 40 ng/mL (area under the curve (AUC) = 0.59) and 150 mAU/mL (AUC = 0.65), respectively. The analysis demonstrated an AUC of 0.65 (95% confidence interval (CI) 0.57–0.73, *p* < 0.0001) for DCP, indicating moderate but clinically meaningful discriminative performance (Figure S1).

For MVI, both AFP > 40 ng/mL (*p* = 0.0070) and DCP > 150 mAU/mL (*p* < 0.0001) were identified as significantly associated factors for its presence in the univariate analysis. In the multivariate logistic regression analysis, DCP > 150 mAU/mL (hazard ratio [HR] = 3.501, 95% CI: 1.892–6.480, *p* < 0.0001) remained an independent predictor for MVI, while AFP did not retain statistical significance (*p* = 0.1687).

Regarding IM, only DCP > 150 mAU/mL (HR = 11.169, 95% CI: 2.321–53.749, *p* = 0.0001) was identified as a significant factor for its presence in the univariate analysis. Since no other variables showed significant associations in the univariate analysis, a multivariate analysis was not performed for IM (Table S2).

Based on these findings, the patients were stratified by their preoperative DCP levels to identify a low‐risk subgroup presumed to be free of MVI and IM. This low‐risk cohort was subjected to comparative analyses between the AR and NAR groups. To eliminate potential bias arising from the tumor burden, we limited the analysis to patients with tumor size ≤ 5 cm, based on our previous finding that tumor size > 5 cm was significantly associated with recurrence after hepatic resection.

### 3.2 Comparison of Surgical and Oncological Outcomes Between the AR and NAR Groups for Low‐Risk Solitary HCC ≤ 5 cm

We focused on patients with solitary HCC measuring ≤ 5 cm who were predicted to be free of MVI and IM based on preoperative DCP ≤ 150 mAU/mL. A total of 214 patients met these criteria and were selected for further analysis. By examining this low‐risk cohort, we sought to determine the true oncological benefit of AR compared with NAR in a clinical setting where the theoretical advantage of AR—complete prevention of microscopic tumor spread via the portal venous system—is presumed to be minimal or negligible. The clinicopathological characteristics and perioperative outcomes of the patients in the low‐risk cohort are shown in Table 1. There were no significant differences between the AR (*n* = 94) and NAR (*n* = 120) groups in terms of age, sex, hepatitis B surface antigen positivity, hepatitis C virus antibody positivity, Child–Pugh classification, liver damage grade, AFP level, DCP level, or tumor differentiation. However, the NAR group had a significantly higher BMI (23.8 vs. 22.8 kg/m^2^, *p* = 0.0089) and a significantly lower albumin level (3.9 vs. 4.1 g/dL, *p* = 0.0215). The NAR group also had a significantly higher proportion of laparoscopic surgery (53.3% vs. 18.1%, *p* < 0.0001). Meanwhile, the AR group had a significantly longer operative time (301 vs. 229 min, *p* < 0.0001) and significantly greater intraoperative blood loss (373 vs. 213 mL, *p* = 0.0003) as well as a significantly longer postoperative hospital stay (13 vs. 10 days, *p* = 0.0157). The median surgical margin was 3.0 mm in the NAR group and 3.5 mm in the AR group (*p* = 0.025), indicating that non‐anatomical resections were not performed with excessively wide margins. The median resected liver weight was 40 mL in the NAR group and 162.5 mL in the AR group (*p* < 0.0001), reflecting the systematic removal of the corresponding portal territory in anatomical resection. In the entire cohort, postoperative complications occurred in 8.8% of laparoscopic cases and 18.7% of open cases (*p* = 0.021), indicating that laparoscopic surgery was associated with a significantly lower complication rate. The R0 resection rate was similarly high in both groups (NAR: 97.5% [117/120] vs. AR: 98.9% [93/94], *p* = 0.4278), indicating that the extent of resection did not affect the likelihood of achieving negative surgical margins. The AR group also had a significantly higher rate of postoperative complications (22.3% vs. 7.6%, *p* = 0.0017).

**TABLE 1:** Comparison of the clinicopathological characteristics and perioperative outcomes between the AR and NAR groups among the low‐risk HCC patients (tumor size ≤ 5 cm, DCP ≤ 150 mAU/mL).

Variable	AR (*n* = 94)	NAR (*n* = 120)	*p*
Age (years)	71 (65–77)	69 (63–76)	0.3318
Male sex	70/24	80/40	0.2141
BMI (kg/m^2^)	22.8 (20.2–25.5)	23.8 (21.4–26.4)	0.0089
HBsAg positive	10 (10.6%)	17 (14.3%)	0.4240
HCVAb positive	52 (55.9%)	69 (57.8%)	0.7627
Albumin (g/dL)	4.1 (3.8–4.3)	3.9 (3.6–4.2)	0.0215
Child–Pugh classification, grade B	1 (1.1%)	3 (2.5%)	0.4278
Liver damage grade A/B	10/84	20/100	0.2557
AFP (ng/mL)	6.9 (3.5–16.8)	6.8 (3.5–32.4)	0.8757
DCP (mAU/mL)	26 (19.5–54.0)	26 (18.0–55.8)	0.9038
TNM Stage (I/II/III/IV)	17/60/17/0	52/63/5/0	< 0.0001
Tumor size (cm)	2.7 (2.0–3.5)	2.0 (1.5–2.5)	< 0.0001
Poor differentiation	17 (18.1%)	26 (21.7%)	0.5150
Microscopic vascular invasion	20 (21.3%)	9 (7.5%)	0.0034
Microscopic intrahepatic metastasis	2 (2.1%)	0 (0%)	0.0687
Liver fibrosis (F3 or F4)	32 (34.0%)	66 (55.9%)	0.0014
Laparoscopic surgery	17 (18.1%)	64 (53.3%)	< 0.0001
Operative time (min)	301 (234–373)	229 (170–284)	< 0.0001
Intraoperative blood loss (mL)	373 (222–720)	213 (71–399)	0.0003
Surgical margin (mm)	3.5 (1.0–10.0)	3.0 (1.0–7.0)	0.0125
Resected liver weight (g)	162 (92–266)	40 (20–69)	< 0.0001
Postoperative hospital stay (days)	13 (10–17)	10 (8–12)	0.0157
In‐hospital mortality	0 (0%)	0 (0%)	—
Postoperative complications	21 (22.3%)	9 (7.6%)	0.0017

*Note:* Data are presented as *n* (%) or median (interquartile range).

Abbreviations: AFP, α‐fetoprotein; AR, anatomical resection; BMI, body mass index; DCP, des‐γ‐carboxyprothrombin; HBsAg, hepatitis B surface antigen; HCC, hepatocellular carcinoma; HCVAb, hepatitis C virus antibody; NAR, non‐anatomical resection.

**FIGURE 1:**
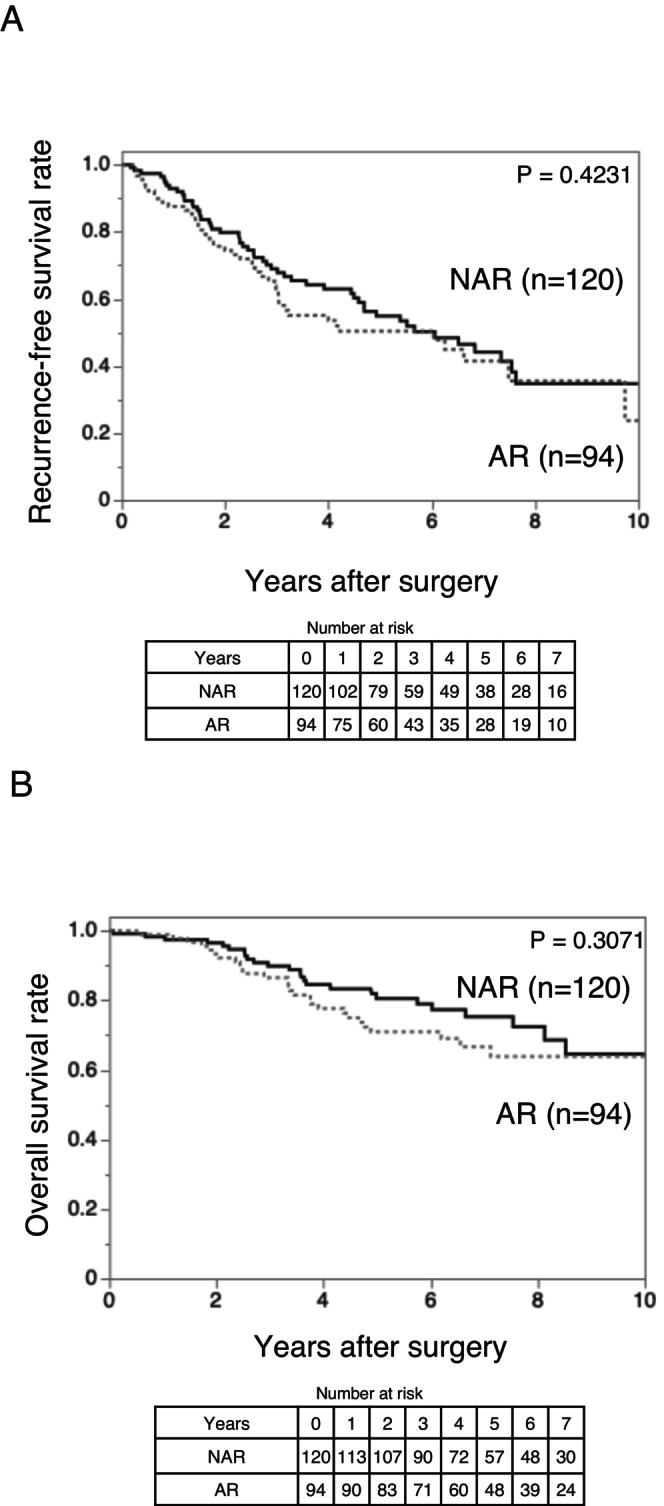
Kaplan–Meier curves comparing recurrence‐free survival (A) and overall survival (B) between patients who underwent AR (*n* = 94) and NAR (*n* = 120) for hepatocellular carcinoma with tumor size ≤ 5 cm and des‐γ‐carboxy prothrombin ≤ 150 mAU/mL. (A) Recurrence‐free survival did not differ significantly between the two groups (*p* = 0.4231). (B) Overall survival also did not differ significantly between the two groups (*p* = 0.3071). AR, anatomical resection; NAR, non‐anatomical resection.

Regarding the oncological outcomes, RFS and OS were comparable between the two groups. Specifically, Kaplan–Meier analysis showed no significant differences in RFS (*p* = 0.4231) and OS (*p* = 0.3071) between the AR and NAR groups (Figure 1).

These findings suggest that, in solitary HCC ≤ 5 cm without predicted MVI and IM, AR does not offer a clear survival advantage over NAR, supporting the feasibility of parenchyma‐preserving surgery in appropriately selected patients.

### 3.3 Propensity Score Matching and Survival Analysis

Propensity score matching was performed using certain preoperative variables, including age, sex, BMI, serum albumin level, Child–Pugh classification, AFP level, DCP level, TNM stage, and maximum tumor diameter, to balance the baseline characteristics between the AR and NAR groups. After the propensity score matching, 41 patients were included in each group, with no significant differences observed in the matched covariates (Table 2).

**TABLE 2:** Baseline characteristics in the AR and NAR groups after propensity score matching among patients with solitary HCC ≤ 5 cm without predicted MVI or IM.

Variable	AR (*n* = 41)	NAR (*n* = 41)	*p*
Age (years)	71 (64–77)	71 (62–78)	0.8372
Male sex	30/11	29/12	0.8058
BMI (kg/m^2^)	22.9 (19.8–25.8)	23.3 (21.0–24.9)	0.5730
HBsAg positive	7 (17.1%)	3 (7.3%)	0.1717
HCVAb positive	25 (61.0%)	69 (58.5%)	0.8218
Albumin (g/dL)	3.9 (3.7–4.3)	3.9 (3.7–4.3)	0.9125
Child–Pugh classification, grade B	0 (0%)	0 (0%)	—
Liver damage grade A/B	4/37	2/39	0.3922
AFP (ng/mL)	7.0 (4.6–27.3)	4.7 (2.6–25.9)	0.5648
DCP (mAU/mL)	27.0 (19.5–44.0)	26.0 (17.0–42.0)	0.7089
TNM Stage (I/II/III/IV)	11/26/4/0	13/24/4/0	0.8839
Tumor size (cm)	2.2 (1.7–2.9)	2.3 (1.7–2.7)	0.9374
Poor differentiation	6 (14.6%)	11 (26.8%)	0.1706
Microscopic vascular invasion	6 (14.6%)	6 (14.6%)	1.0000
Microscopic intrahepatic metastasis	0 (0%)	0 (0%)	—
Liver fibrosis (F3 or F4)	15 (36.6%)	20 (48.8%)	0.3192
Laparoscopic surgery	13 (31.7%)	24 (58.5%)	0.0140
Operative time (min)	301 (238–360)	213 (170–271)	0.0002
Intraoperative blood loss (mL)	361 (240–817)	180 (40–335)	0.0010
Postoperative hospital stay (days)	12 (10–17)	10 (8–12)	0.0027
In‐hospital mortality	0 (0%)	0 (0%)	—
Postoperative complications	10 (24.4%)	3 (7.3%)	0.0300

*Note:* Data are presented as *n* (%) or median (interquartile range).

Abbreviations: AFP, α‐fetoprotein; AR, anatomical resection; BMI, body mass index; DCP, des‐γ‐carboxyprothrombin; HBsAg, hepatitis B surface antigen; HCC, hepatocellular carcinoma; HCVAb, hepatitis C virus antibody; IM, intrahepatic metastasis; MVI, microvascular invasion; NAR, non‐anatomical resection.

In the matched cohort, the AR group had a significantly longer operative time (301 vs. 213 min, *p* = 0.0002) and significantly greater intraoperative blood loss (361 vs. 180 mL, *p* = 0.0010) compared with the NAR group. The R0 resection rate remained identical between the two groups (97.6% in both AR and NAR). The AR group also had a significantly longer postoperative hospital stay (12 vs. 10 days, *p* = 0.0027) and a significantly higher rate of postoperative complications (24.4% vs. 7.3%, *p* = 0.0300). There were no in‐hospital deaths in either group.

**FIGURE 2:**
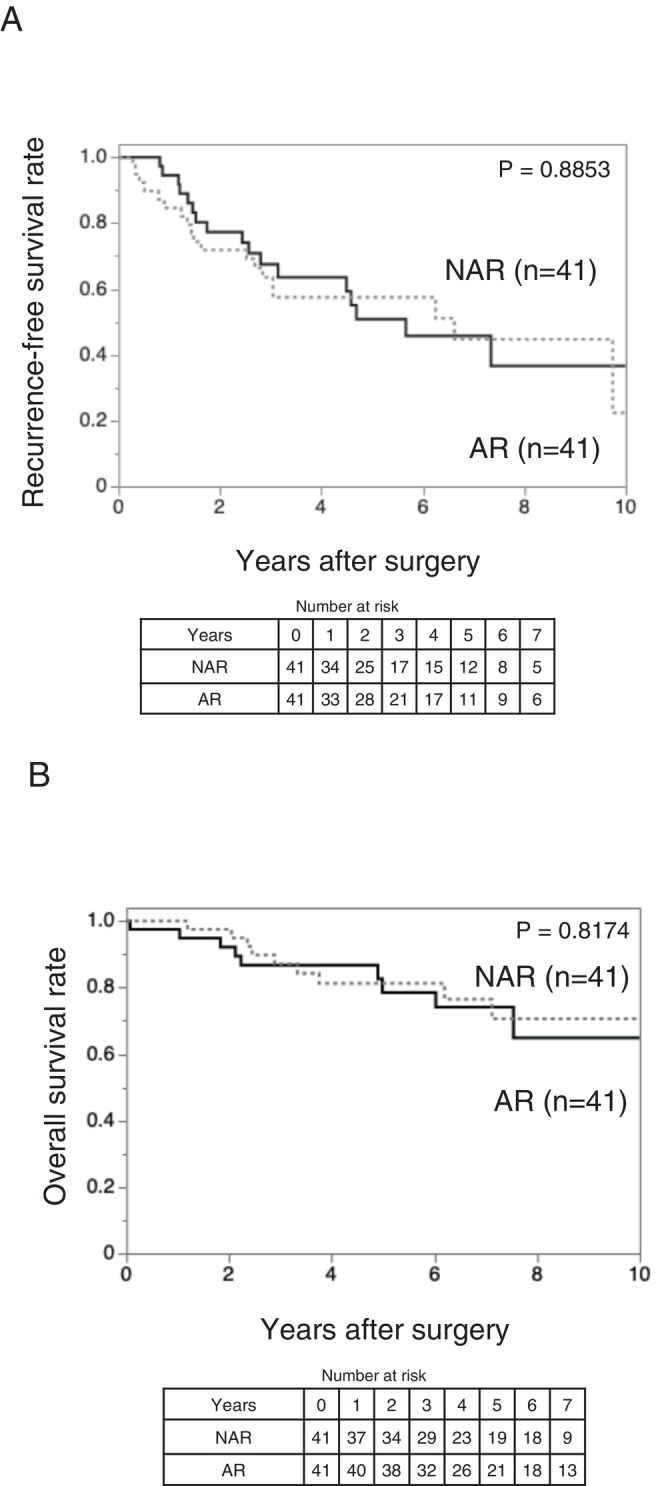
Kaplan–Meier curves comparing recurrence‐free survival (A) and overall survival (B) between the AR (*n* = 41) and NAR (*n* = 41) groups after propensity score matching in patients with tumor size ≤ 5 cm and des‐γ‐carboxy prothrombin ≤ 150 mAU/mL. (A) Recurrence‐free survival did not differ significantly between the two groups (*p* = 0.8853). (B) Overall survival also did not differ significantly between the two groups (*p* = 0.8174). AR, anatomical resection; NAR, non‐anatomical resection.

Regarding the oncological outcomes, Kaplan–Meier analysis demonstrated no significant difference in RFS between the AR and NAR groups (*p* = 0.8853) (Figure 2A). Similarly, OS did not differ significantly between the two groups (*p* = 0.8174) (Figure 2B). These findings suggest that, after balancing the preoperative background characteristics, AR does not confer a significant advantage over NAR in terms of RFS or OS in patients with solitary HCC ≤ 5 cm without predicted MVI and IM.

For completeness, survival outcomes in the entire 303‐patient cohort and in the high‐risk subgroup with DCP > 150 mAU/mL were also compared between AR and NAR, and no significant differences were observed (Figures S2 and S3).

### 3.4 Prognostic Comparison Between the AR and NAR Groups for Low‐Risk Solitary HCC Stratified by Tumor Size

**FIGURE 3:**
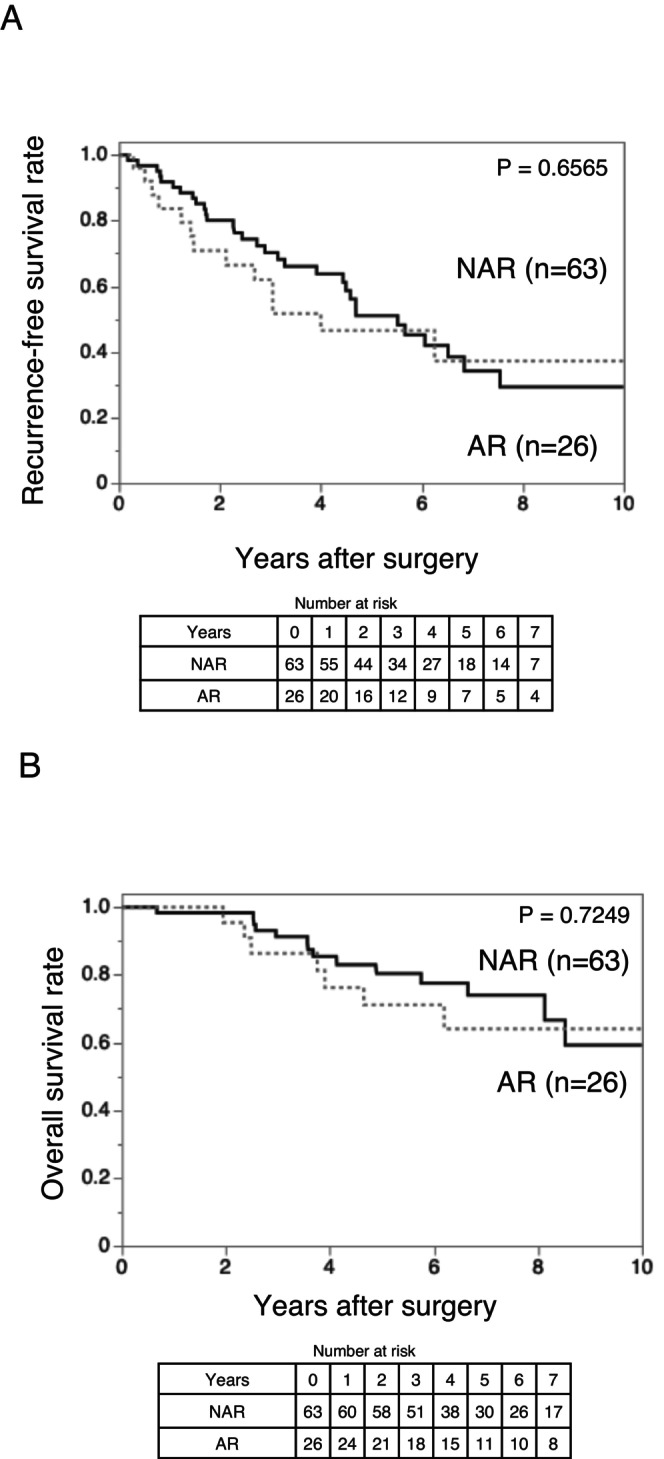
Kaplan–Meier curves comparing (A) recurrence‐free survival and (B) overall survival between the AR (*n* = 26) and NAR (*n* = 63) groups for patients with tumor size 0–2.0 cm and des‐γ‐carboxy prothrombin ≤ 150 mAU/mL. Both recurrence‐free survival (*p* = 0.6565) and overall survival (*p* = 0.7249) did not differ significantly between the groups. AR, anatomical resection; NAR, non‐anatomical resection.

In the 0–2.0 cm subgroup (Table 3), the AR group had a slightly higher proportion of stage III tumors than the NAR group, although the absolute number of stage III cases was small in both groups. Severe liver fibrosis was more frequent in the NAR group. Kaplan–Meier analysis revealed no significant differences in RFS (*p* = 0.6565) and OS (*p* = 0.7249) between the AR and NAR groups (Figure 3). These findings suggest that, for solitary HCC ≤ 2 cm without predicted MVI and IM, NAR achieves survival outcomes comparable to those obtained after AR.

**TABLE 3:** Baseline characteristics of the patients with solitary HCC measuring 0–2.0 cm without predicted MVI and IM: Comparison between the AR and NAR groups.

Variable	AR (*n* = 26)	NAR (*n* = 63)	*p*
Age (years)	71 (68–77)	67 (63–75)	0.0808
Male sex	19/7	40/23	0.3788
BMI (kg/m^2^)	23.0 (20.2–25.6)	24.2 (21.7–26.5)	0.0850
HBsAg positive	4 (15.4%)	8 (12.7%)	0.7592
HCVAb positive	20 (77.0%)	41 (65.1%)	0.2654
Albumin (g/dL)	3.9 (3.8–4.3)	3.9 (3.6–4.1)	0.3358
Child–Pugh classification, grade B	0 (0%)	1 (1.6%)	0.4042
Liver damage grade A/B	4/22	52/11	0.7868
AFP (ng/mL)	7.1 (4.7–18.6)	8.1 (3.7–48.9)	0.3169
DCP (mAU/mL)	23.0 (17.8–35.0)	27.0 (17.0–55.0)	0.0838
TNM Stage (I/II/III/IV)	17/6/3/0	52/11/0/0	0.0150
Tumor size (cm)	1.6 (1.4–1.8)	1.5 (1.1–1.7)	0.1934
Poor differentiation	2 (7.7%)	15 (23.8%)	0.0596
Microscopic vascular invasion	6 (23.1%)	4 (6.4%)	0.0307
Microscopic intrahepatic metastasis	0 (0%)	0 (0%)	—
Liver fibrosis (F3 or F4)	10 (38.5%)	41 (65.1%)	0.0212
Laparoscopic surgery	8 (30.8%)	34 (54.0%)	0.0438
Operative time (min)	311 (227–372)	222 (170–271)	< 0.0001
Intraoperative blood loss (mL)	437 (174–902)	195 (50–400)	0.0005
Surgical margin (mm)	3.0 (0.8–10.0)	3.0 (1.0–8.0)	0.1357
Resected liver weight (g)	179 (91–263)	36 (16–59)	< 0.0001
Postoperative hospital stay (days)	12 (11–16)	10 (8–12)	0.0080
In‐hospital mortality	0 (0%)	0 (0%)	—
Postoperative complications	6 (23.1%)	7 (12.1%)	0.2095

*Note:* Data are presented as *n* (%) or median (interquartile range).

Abbreviations: AFP, α‐fetoprotein; AR, anatomical resection; BMI, body mass index; DCP, des‐γ‐carboxyprothrombin; HBsAg, hepatitis B surface antigen; HCC, hepatocellular carcinoma; HCVAb, hepatitis C virus antibody; IM, intrahepatic metastasis; MVI, microvascular invasion; NAR, non‐anatomical resection.

**FIGURE 4:**
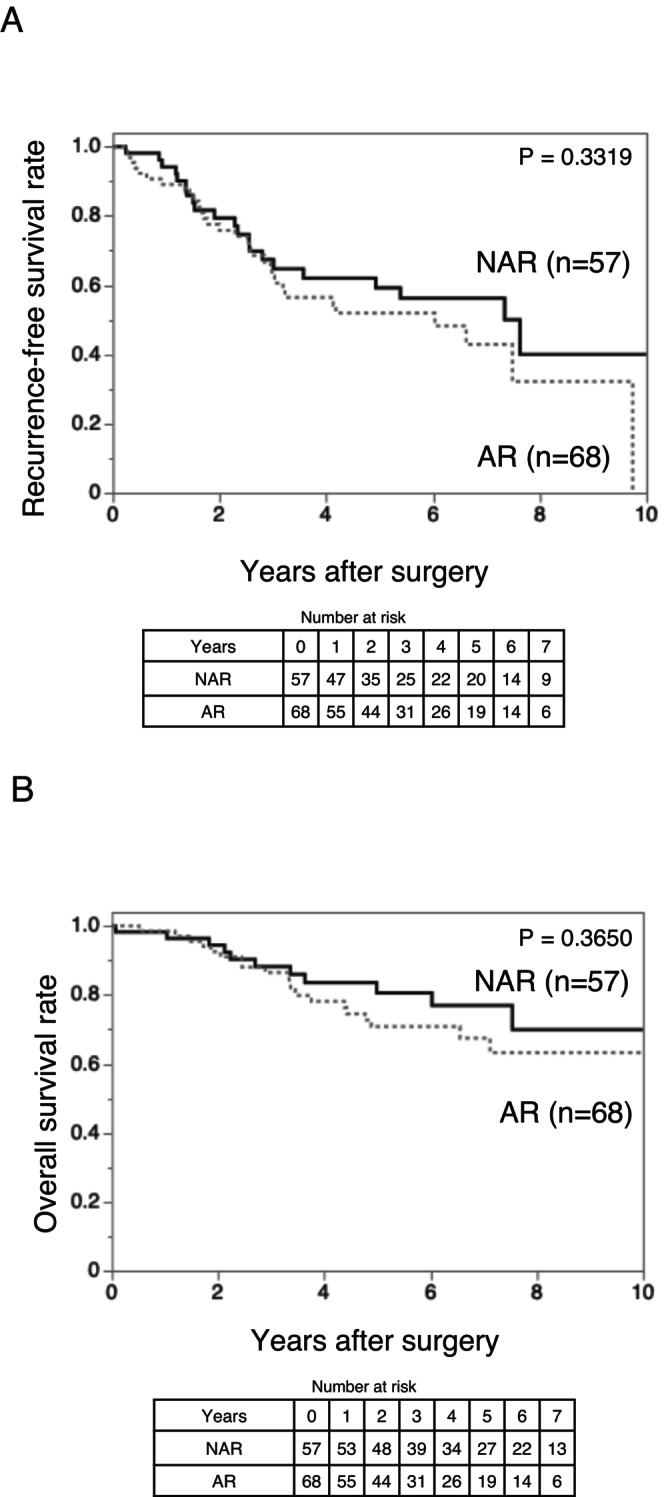
Kaplan–Meier curves comparing (A) recurrence‐free survival and (B) overall survival between the AR (*n* = 68) and NAR (*n* = 57) groups for patients with tumor size 2.1–5.0 cm and des‐γ‐carboxy prothrombin ≤ 150 mAU/mL. Neither recurrence‐free survival (*p* = 0.3319) nor overall survival (*p* = 0.3650) showed a significant difference between the groups. AR, anatomical resection; NAR, non‐anatomical resection.

In the 2.1–5.0 cm subgroup (Table 4), the baseline characteristics were generally balanced between the AR and NAR groups, and the RFS (*p* = 0.3319) and OS (*p* = 0.3650) were similar (Figure 4). This consistency across the subgroups supports the use of parenchyma‐preserving NAR in low‐risk patients—defined as solitary HCC ≤ 5 cm with preoperative DCP ≤ 150 mAU/mL and predicted absence of MVI and IM.

**TABLE 4:** Baseline characteristics of the patients with solitary HCC measuring 2.1–5.0 cm without predicted MVI and IM: Comparison between the AR and NAR groups.

Variable	AR (*n* = 68)	NAR (*n* = 57)	*p*
Age (years)	71 (64–77)	72 (61–77)	0.8543
Male sex	51/17	40/17	0.5465
BMI (kg/m^2^)	22.6 (20.0–25.4)	23.8 (20.6–26.4)	0.0860
HBsAg positive	6 (8.6%)	9 (15.8%)	0.2332
HCVAb positive	32 (47.1%)	28 (49.1%)	0.8046
Albumin (g/dL)	4.1 (3.8–4.3)	3.9 (3.6–4.3)	0.0869
Child–Pugh classification, grade B	1 (1.5%)	2 (3.5%)	0.4572
Liver damage grade A/B	6/62	8/49	0.3584
AFP (ng/mL)	6.8 (3.0–16.2)	5.8 (2.9–23.5)	0.3641
DCP (mAU/mL)	34.5 (21.0–62.8)	26.0 (18.5–57.0)	0.5589
TNM Stage (I/II/III/IV)	0/54/14/0	0/52/5/0	0.0611
Tumor size (cm)	3.0 (2.5–3.8)	2.5 (2.3–3.2)	0.0010
Poor differentiation	15 (22.1%)	11 (19.3%)	0.7044
Microscopic vascular invasion	14 (20.6%)	5 (8.8%)	0.0611
Microscopic intrahepatic metastasis	2 (1.6%)	0 (0%)	0.1166
Liver fibrosis (F3 or F4)	22 (32.4%)	25 (43.9%)	0.1372
Laparoscopic surgery	9 (13.2%)	30 (52.6%)	< 0.0001
Operative time (min)	301 (238–387)	234 (170–289)	< 0.0001
Intraoperative blood loss (mL)	361 (230–571)	239 (100–386)	0.0349
Surgical margin (mm)	4.0 (1.0–10.0)	2.0 (1.0–6.0)	0.0647
Resected liver weight (g)	158 (92–294)	44 (25–88)	< 0.0001
Postoperative hospital stay (days)	14 (10–18)	10 (8–14)	0.0930
In‐hospital mortality	0 (0%)	0 (0%)	—
Postoperative complications	15 (22.1%)	2 (3.6%)	0.0008

*Note:* Data are presented as *n* (%) or median (interquartile range).

Abbreviations: AFP, α‐fetoprotein; AR, anatomical resection; BMI, body mass index; DCP, des‐γ‐carboxyprothrombin; HBsAg, hepatitis B surface antigen; HCC, hepatocellular carcinoma; HCVAb, hepatitis C virus antibody; IM, intrahepatic metastasis; MVI, microvascular invasion; NAR, non‐anatomical resection.

## 4 Discussion

In HCC surgery, two main resection approaches are employed: AR, which removes the entire portal venous territory of the tumor‐bearing segment, and NAR, which preserves the uninvolved parenchyma. AR is theoretically advantageous in eradicating MVI and IM through complete removal of the portal venous drainage area [8]. However, in low‐risk cases—such as solitary tumors with a small size and no MVI or IM—the extent to which this theoretical benefit translates into improved long‐term outcomes remains unclear. According to the 2023 Japanese Expert Consensus Statement on Oncological Resectability, solitary tumors without MVI or IM are classified as resectable, and surgical resection is recommended in such cases [5]. Our previous study demonstrated that tumor size > 5 cm was associated with a significantly increased risk of recurrence, providing a scientific rationale for limiting the present analysis to patients with tumor size ≤ 5 cm [6]. In the present study, we first identified preoperative DCP ≤ 150 mAU/mL as a significant predictor for the absence of MVI and IM. We then focused on patients with solitary HCC ≤ 5 cm who met this DCP criterion and compared the oncological outcomes between the AR and NAR groups. Our findings showed no significant differences in RFS or OS between the two approaches in this low‐risk cohort, suggesting that parenchyma‐preserving NAR may be an acceptable alternative when the likelihood of MVI and IM is minimal.

Previous studies have provided conflicting findings regarding the superiority of AR over NAR for small HCCs. A large retrospective analysis demonstrated that AR offered significantly better OS and RFS than NAR for HCC < 5 cm, while showing no benefit for larger tumors after propensity score matching [9]. Yamashita et al. [10] indicated that AR should be recommended for noncirrhotic patients with HCC and preserved liver function (liver damage grade A). For patients with solitary HCC ≤ 5 cm, Shindo et al. [11] showed that AR prolonged RFS compared with NAR, but was not associated with improved OS. Among patients with initial HCC, Marubashi et al. [12] found no differences in OS and RFS after NAR or AR. While multiple meta‐analyses and large‐scale observational studies have reported superior survival outcomes for AR, particularly in patients with high‐risk features [13, 14, 15], these cohorts frequently included a substantial proportion of patients with MVI or IM, both of which are strong predictors of recurrence and poor prognosis. However, few studies have specifically examined the outcomes in low‐risk patients without MVI and IM. To our knowledge, the present study is the first to focus exclusively on this low‐risk subgroup, enabling a direct assessment of whether the theoretical advantage of AR translates into an actual survival benefit when the likelihood of microscopic spread is presumed to be minimal.

In our propensity score‐matched analysis based on preoperative variables only, we found no significant differences in RFS or OS between the AR and NAR groups within the low‐risk cohort. Operative time and blood loss were greater in the AR group, but the differences did not translate into improved oncological outcomes. Similarly, the subgroup analyses by tumor size (0–2.0 and 2.1–5.0 cm) demonstrated no survival benefit for AR over NAR. Although a slightly higher proportion of stage III tumors was present in the AR group within the 0–2.0 cm subgroup, the absolute numbers were small, and this imbalance is unlikely to have influenced the OS results. Furthermore, postoperative complications were significantly less frequent after laparoscopic surgery than after open surgery in the overall cohort (8.8% vs. 18.7%, *p* = 0.021). Since the proportion of laparoscopic procedures was lower in the AR group, the higher complication rate observed in this group may be partly attributable to the surgical approach rather than the extent of resection itself.

The present findings have important clinical implications. In patients with solitary HCC ≤ 5 cm, preserved liver function, and low predicted risk of MVI and IM, NAR appears to be an acceptable alternative to AR, allowing for maximal preservation of the functional liver parenchyma without compromising the long‐term outcomes. This approach may be particularly relevant in the era of increasing repeat hepatectomies and multimodal treatment strategies, where conservation of the liver reserve is crucial. By focusing on a biologically favorable subset of HCC patients, our study underscores the importance of tailoring the extent of resection to the patient's oncological risk profile rather than applying a uniform surgical strategy.

This study has several limitations. First, its retrospective single‐institution design introduces the potential for selection bias, even though we performed propensity score matching using preoperative factors. Importantly, postoperative pathological findings, such as tumor differentiation, MVI, and IM, were intentionally excluded from the matching process, because they were not available preoperatively and would introduce post‐treatment bias. Second, although DCP showed predictive value for MVI and IM, its diagnostic accuracy was not absolute, and some patients classified as low risk may have harbored microscopic disease. In addition, although NAR may theoretically miss small IM due to its limited resection volume, this bias is unlikely to have affected the identification of preoperative predictors in this study because MVI is assessed adjacent to the main tumor and DCP > 150 mAU/mL remained the only independent predictor of both MVI and IM. Finally, external validation in diverse patient populations, including those outside Japan, is warranted before generalization of our findings.

## Author Contributions

**Norifumi Iseda:** conceptualization, methodology, data curation, investigation, formal analysis, writing – original draft, writing – review and editing. **Shinji Itoh:** conceptualization, methodology, data curation, writing – review and editing, investigation, funding acquisition, formal analysis. **Kyohei Yugawa:** investigation. **Shohei Yoshiya:** investigation. **Takashi Motomura:** investigation. **Takeo Toshima:** investigation. **Tomoharu Yoshizumi:** supervision, writing – review and editing, formal analysis.

## Funding

This study was supported by JSPS KAKENHI (grant number: JP‐23K08133).

## Ethics Statement

This study was approved by the Ethics Committee of Kyushu University (approval code: 2021–468).

## Consent

Informed consent was obtained from all patients.

## Conflicts of Interest

The authors declare no conflicts of interest for this article. Dr. Tomoharu Yoshizumi is an Editorial Board Member of *Annals of Gastroenterological Surgery*.

## Supporting information


**Figure S1:** Receiver operating characteristic (ROC) curve of des‐γ‐carboxy prothrombin (DCP) for predicting microvascular invasion (MVI) in patients with hepatocellular carcinoma (HCC).
**Figure S2:** Recurrence‐free survival (A) and overall survival (B) in the entire cohort of 303 patients with solitary hepatocellular carcinoma, comparing anatomical resection and non‐anatomical resection.
**Figure S3:** Recurrence‐free survival (A) and overall survival (B) in high‐risk patients with solitary hepatocellular carcinoma defined by preoperative des‐γ‐carboxy prothrombin > 150 mAU/mL, comparing anatomical resection (AR) and non‐anatomical resection (NAR).

**Table S1:** Clinicopathological characteristics of the patients.

**Table S2:** Univariate and multivariate analyses of preoperative factors associated with intrahepatic metastasis and microvascular invasion.
