# Supplementary material for: Comparison of Anatomical and Non‐Anatomical Resection in Low Microvascular Invasion Risk Solitary Hepatocellular Carcinoma ≤ 5 cm
Source: Ann Gastroenterol Surg. 2025 Dec 26;10(3):861–70. doi: 10.1002/ags3.70157 (PMC13178266; doi:10.1002/ags3.70157)
Supplement: Supplementary file 1 — Figure S1: Receiver operating characteristic (ROC) curve of des‐γ‐carboxy prothrombin (DCP) for predicting microvascular invasion (MVI) in patients with hepatocellular carcinoma (HCC). Figure S2: Recurrence‐free survival (A) and overall survival (B) in the entire cohort of 303 patients with solitary hepatocellular carcinoma, comparing anatomical resection and non‐anatomical resection. Figure S3: Recurrence‐free survival (A) and overall survival (B) in high‐risk patients with solitary hepatocellular carcinoma defined by preoperative des‐γ‐carboxy prothrombin > 150 mAU/mL, comparing anatomical resection (AR) and non‐anatomical resection (NAR). [file AGS3-10-861-s002.pptx]

## Slide 1
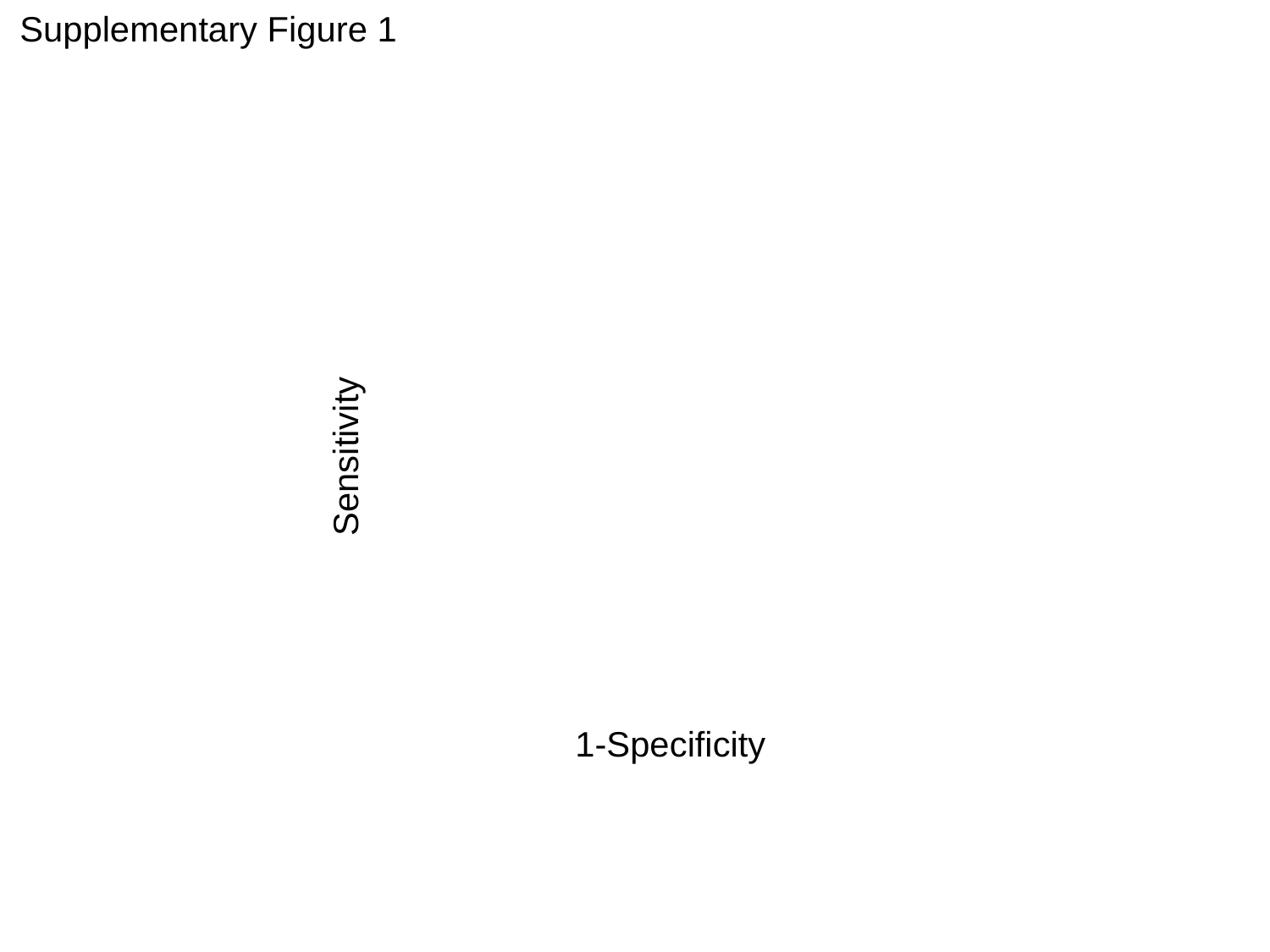

Supplementary Figure 1
Sensitivity
1-Specificity

## Slide 2
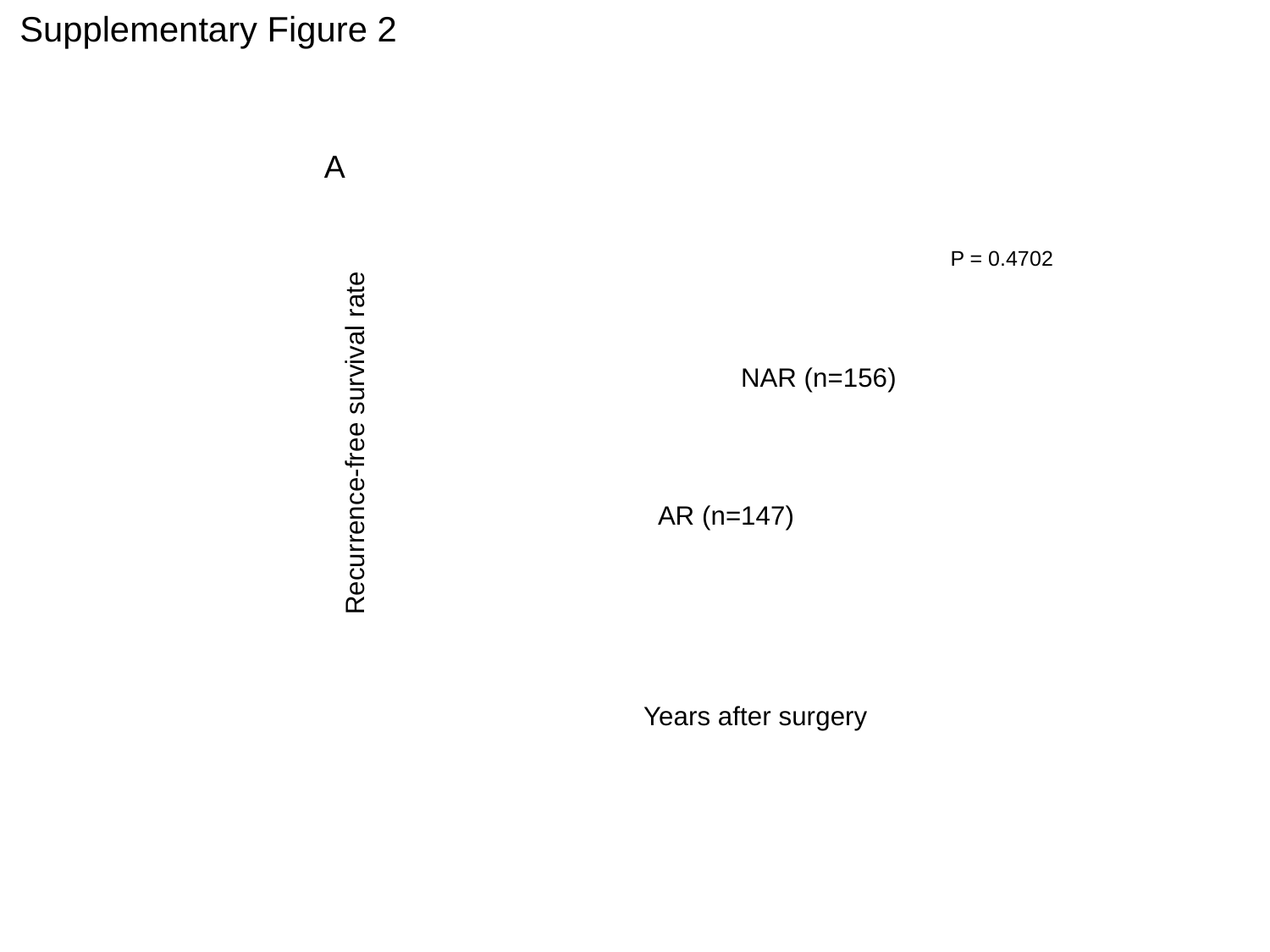

Supplementary Figure 2
A
P = 0.4702
NAR (n=156)
Recurrence-free survival rate
AR (n=147)
 Years after surgery

## Slide 3
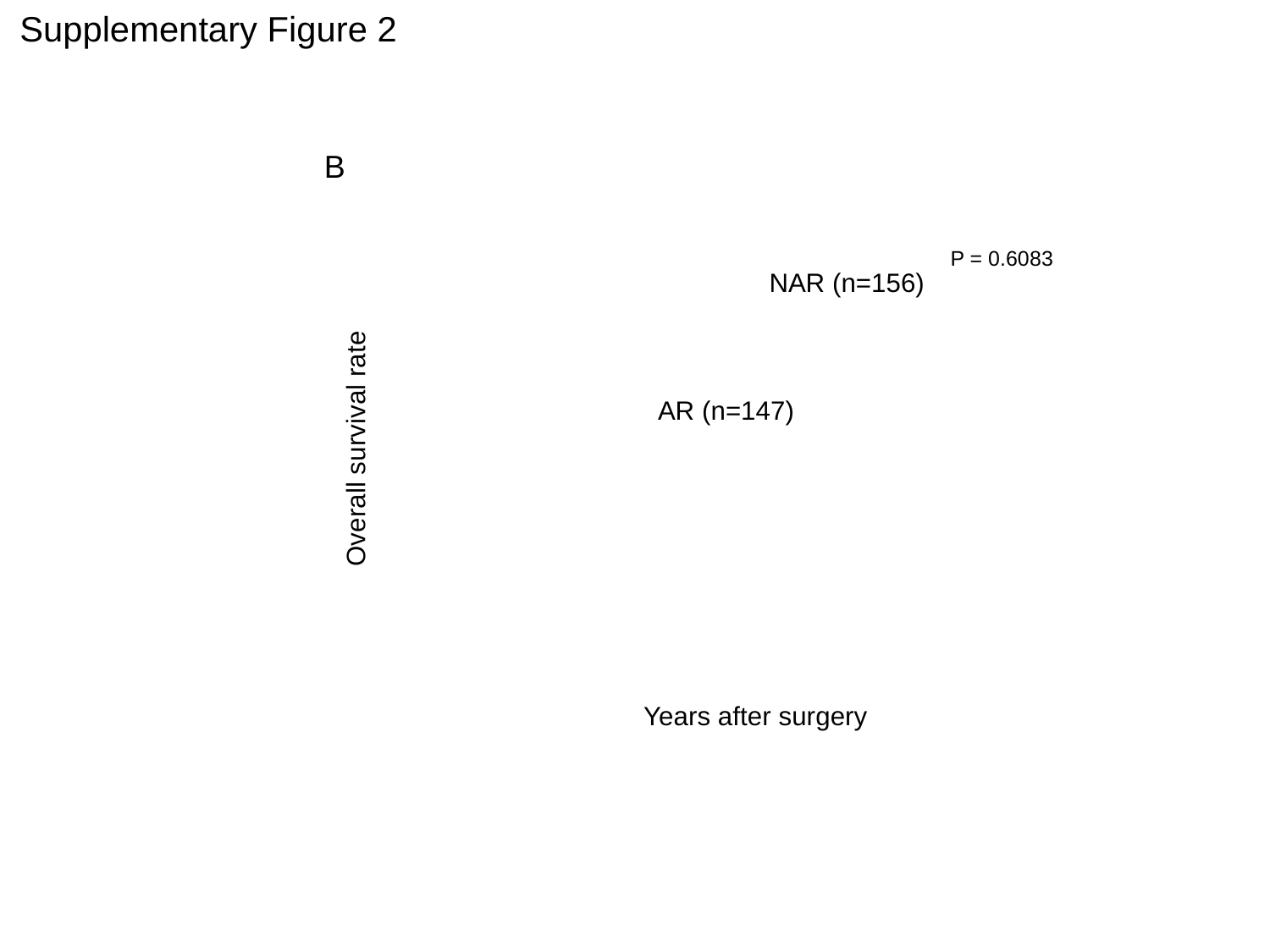

Supplementary Figure 2
B
P = 0.6083
NAR (n=156)
AR (n=147)
Overall survival rate
 Years after surgery

## Slide 4
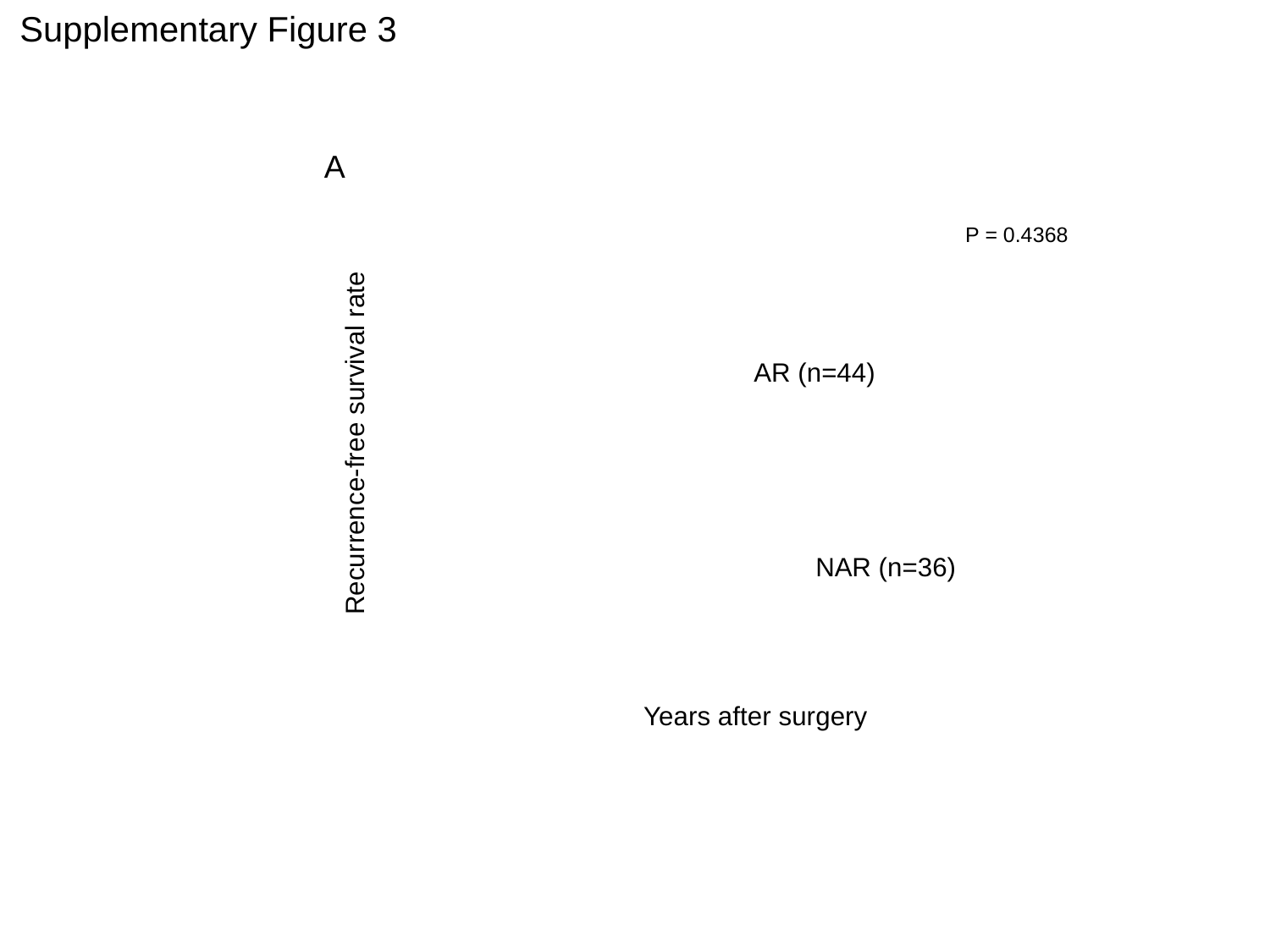

Supplementary Figure 3
A
P = 0.4368
AR (n=44)
Recurrence-free survival rate
NAR (n=36)
 Years after surgery

## Slide 5
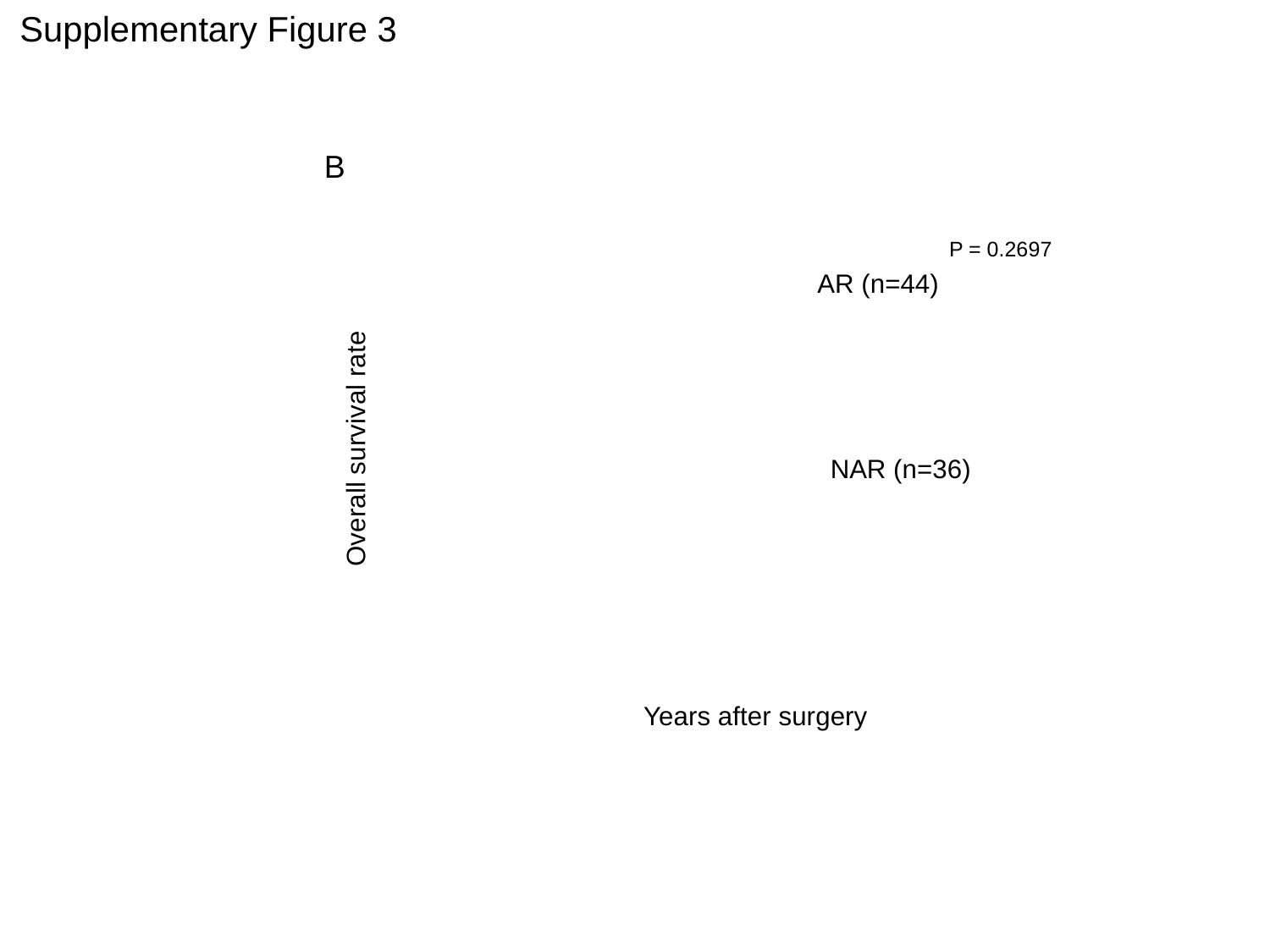

Supplementary Figure 3
B
P = 0.2697
AR (n=44)
Overall survival rate
NAR (n=36)
 Years after surgery
