# Supplementary material for: Comparison of Anatomical and Non‐Anatomical Resection in Low Microvascular Invasion Risk Solitary Hepatocellular Carcinoma ≤ 5 cm
Source: Ann Gastroenterol Surg. 2025 Dec 26;10(3):861–70. doi: 10.1002/ags3.70157 (PMC13178266; doi:10.1002/ags3.70157)
Supplement: Supplementary file 2 — Table S1: Clinicopathological characteristics of the patients. [file AGS3-10-861-s003.docx]

Supplementary Table 1. Clinicopathological characteristics of the patients

| Variable |  |
| --- | --- |
| Age (years) | 69 (63–75) |
| Male sex | 215/88 |
| BMI (kg/m²) | 23.2 (20.8–25.5) |
| HBsAg positive | 47 (15.5%) |
| HCVAb positive | 164 (54.1%) |
| Albumin (g/dL) | 4.0 (3.7-4.3) |
| Child–Pugh classification, grade B | 5 (1.7%) |
| Liver damage grade A / B | 268/35 |
| AFP (ng/mL) | 8.7 (3.9-44.0) |
| DCP (mAU/mL) | 46 (23.0–183.3) |
| TNM Stage (I / II / III / IV) | 75/176/52/0 |
| Tumor size (cm) | 2.5 (1.8–3.4) |
| Poor differentiation | 77 (25.4%) |
| Microscopic vascular invasion | 62 (20.5%) |
| Microscopic intrahepatic metastasis | 10 (3.3%) |
| Liver fibrosis (F3 or F4) | 126 (41.6%) |

Data are presented as *n* (%) or median (interquartile range).

BMI, body mass index; HBsAg, hepatitis B surface antigen; HCVAb, hepatitis C virus antibody; AFP, α-fetoprotein; DCP, des-γ-carboxyprothrombin.
