# Supplementary material for: Comparison of Anatomical and Non‐Anatomical Resection in Low Microvascular Invasion Risk Solitary Hepatocellular Carcinoma ≤ 5 cm
Source: Ann Gastroenterol Surg. 2025 Dec 26;10(3):861–70. doi: 10.1002/ags3.70157 (PMC13178266; doi:10.1002/ags3.70157)
Supplement: Supplementary file 3 — Table S2: Univariate and multivariate analyses of preoperative factors associated with intrahepatic metastasis and microvascular invasion. [file AGS3-10-861-s001.docx]

Supplementary Table 2. Univariate and multivariate analyses of preoperative factors associated with intrahepatic metastasis and microvascular invasion

| **Factors** |  | **Intrahepatic metastasis** | |  | **Microvascular invasion** | |  |
| --- | --- | --- | --- | --- | --- | --- | --- |
|  |  | **Univariate analysis** | **Multivariate analysis** |  | **Univariate analysis** | **Multivariate analysis** |  |
|  |  | **Hazard ratio (95% CI)** | **Hazard ratio (95% CI)** |  | **Hazard ratio (95% CI)** | **Hazard ratio**  **(95% CI)** |  |
|  |  | ***P*-value** | ***P*-value** |  | ***P*-value** | ***P*-value** |  |
| Age (years) |  | 0.9640  (0.915–1.016) |  |  | 0.9802  (0.955–1.006) |  |  |
|  |  | 0.1905 |  |  | 0.1343 |  |  |
|  |  |  |  |  |  |  |  |
| Sex | Male | 3.801  (0.474–30.459) |  |  | 1.362  (0.716–2.590) |  |  |
|  | Female | 0.1374 |  |  | 0.3391 |  |  |
|  |  |  |  |  |  |  |  |
| HBsAg | Positive | 3056  (0 – -) |  |  | 1.830  (0.909–3.682) |  |  |
|  | Negative | 0.9984 |  |  | 0.0906 |  |  |
|  |  |  |  |  |  |  |  |
| HCV-Ab | Positive | 0.343  (0.087–1.354) |  |  | 0.856  (0.489–1.499) |  |  |
|  | Negative | 0.1091 |  |  | 0.5881 |  |  |
|  |  |  |  |  |  |  |  |
| Child-Pugh classification | B | 8.028  (0.813–79.236) |  |  | 0.971  (0.107–8.848) |  |  |
|  | A | 0.1414 |  |  | 0.9793 |  |  |
|  |  |  |  |  |  |  |  |
| AFP > 40 ng/mL |  | 1.981  (0.544–7.218) |  |  | 2.276  (1.252–4.137) | 1.571  (0.826–2.991) |  |
|  |  | 0.2997 |  |  | 0.0070 | 0.1687 |  |
|  |  |  |  |  |  |  |  |
| DCP > 150 mAU/mL |  | 11.169  (2.321–53.749) |  |  | 3.914  (2.174–7.045) | 3.501  (1.892–6.480) |  |
|  |  | 0.0001 |  |  | < 0.0001 | < 0.0001 |  |
|  |  |  |  |  |  |  |  |

CI, confidence interval; HBsAg, hepatitis B surface antigen; HCVAb, hepatitis C virus antibody; AFP, α-fetoprotein; DCP, des-γ-carboxyprothrombin.
